# Moderate excess alcohol consumption and adverse cardiac remodelling in dilated cardiomyopathy

**DOI:** 10.1136/heartjnl-2021-319418

**Published:** 2021-08-11

**Authors:** Upasana Tayal, John Gregson, Rachel Buchan, Nicola Whiffin, Brian P Halliday, Amrit Lota, Angharad M Roberts, A John Baksi, Inga Voges, Julian W E Jarman, Resham Baruah, Michael Frenneaux, John G F Cleland, Paul Barton, Dudley J Pennell, James S Ware, Stuart A Cook, Sanjay K Prasad

**Affiliations:** 1 National Heart and Lung Institute, Imperial College London, London, UK; 2 Royal Brompton Hospital, London, UK; 3 London School of Hygiene and Tropical Medicine, London, UK; 4 Medical Research Council Clinical Sciences Centre, Imperial College London, London, UK; 5 Robertson Centre for Biostatistics, University of Glasgow, Glasgow, UK; 6 Duke NUS, Singapore

**Keywords:** cardiomyopathy, dilated, magnetic resonance imaging

## Abstract

**Objective:**

The effect of moderate excess alcohol consumption is widely debated and has not been well defined in dilated cardiomyopathy (DCM). There is need for a greater evidence base to help advise patients. We sought to evaluate the effect of moderate excess alcohol consumption on cardiovascular structure, function and outcomes in DCM.

**Methods:**

Prospective longitudinal observational cohort study. Patients with DCM (n=604) were evaluated for a history of moderate excess alcohol consumption (UK government guidelines; >14 units/week for women, >21 units/week for men) at cohort enrolment, had cardiovascular magnetic resonance and were followed up for the composite endpoint of cardiovascular death, heart failure and arrhythmic events. Patients meeting criteria for alcoholic cardiomyopathy were not recruited.

**Results:**

DCM patients with a history of moderate excess alcohol consumption (n=98, 16%) had lower biventricular function and increased chamber dilatation of the left ventricle, right ventricle and left atrium, as well as increased left ventricular hypertrophy compared with patients without moderate alcohol consumption. They were more likely to be male (alcohol excess group: n=92, 94% vs n=306, 61%, p=<0.001). After adjustment for biological sex, moderate excess alcohol was not associated with adverse cardiac structure. There was no difference in midwall myocardial fibrosis between groups. Prior moderate excess alcohol consumption did not affect prognosis (HR 1.29, 95% CI 0.73 to 2.26, p=0.38) during median follow-up of 3.9 years.

**Conclusion:**

DCM patients with moderate excess alcohol consumption have adverse cardiac structure and function at presentation, but this is largely due to biological sex. Alcohol may contribute to sex-specific phenotypic differences in DCM. These findings help to inform lifestyle discussions for patients with DCM.

## Introduction

The effect of moderate alcohol consumption is widely debated and has not been well defined in dilated cardiomyopathy (DCM). From a global health perspective, there has been increased recent interest in the effects of alcohol on the heart, particularly the potentially beneficial effects of low to moderate alcohol consumption on cardiovascular disease.^1^ Studies indicate that modest alcohol consumption may be associated with a lower risk for heart failure.^2^ However, Mendelian methodological approaches have raised doubts regarding the cardioprotective effects of low to moderate alcohol consumption.^3^ Guidance on alcohol consumption is frequently requested by patients with no clear evidence base to advise patients.

It is established that chronic excess alcohol consumption can lead to an alcoholic cardiomyopathy with adverse outcomes.^4^ However, the effect of a prior history of moderate excess alcohol consumption remains an important unanswered question for clinicians and patients.

In this study, our aim was to determine the effects of a history of moderate excess alcohol consumption on cardiovascular structure, function and outcomes in a well-characterised cohort of patients with DCM. Notably, no patients with a diagnosis of alcoholic cardiomyopathy were recruited to this study.

## Methods

### Data sharing

The data and analysis methods that support the findings of this study are available from the corresponding author (UT) on reasonable request. Data will be shared after review and approval by our Biobank scientific board, and terms of collaboration will be reached together with a signed data access agreement.

### Study population

The study population comprised 604 patients with DCM confirmed by late gadolinium enhancement (LGE) cardiovascular magnetic resonance (CMR) prospectively enrolled in the National Institute for Health Research Royal Brompton Hospital Cardiovascular Biobank project between 2009 and 2015. Patients were recruited from a network of >30 regional hospitals. No patients with a diagnosis of alcoholic cardiomyopathy were recruited (alcohol consumption in excess of 80 g/day for 5 years.^4^ Patients were enrolled at the time of the first diagnostic imaging study. All patients underwent baseline clinical evaluation, ECG, genetic assessment and CMR including evaluation for LGE midwall fibrosis as previously described.^5^ Socioeconomic status was assessed using the Index of Multiple Deprivation quintile. All patients provided written informed consent. The study was approved by the regional ethics committee.

DCM was diagnosed based on established CMR criteria of left ventricular dilation and reduced ejection fraction with reference to age and gender adjusted nomograms^6^ in the absence of known coronary artery disease (presence of subendocardial LGE suggestive of previous myocardial infarction, >50% stenosis in one or more major epicardial coronary arteries or need for previous percutaneous coronary intervention or coronary artery bypass grafting), abnormal loading conditions (uncontrolled hypertension or significant primary valvular disease), acute myocarditis or non-alcohol toxin exposure. A history of well-controlled hypertension or diabetes was documented as comorbidities.

### Alcohol history

Alcohol consumption prior to the diagnosis of DCM was assessed by patient interview and review of medical records. Patient interview using a standardised questionnaire was conducted by a research nurse at study enrolment, and patients’ current weekly alcohol consumption as well as previous alcohol history was recorded. The hospital and primary care medical records were reviewed by the study investigators for any evidence of documented alcohol consumption greater than the threshold for moderate excess (table 1). Both sources of data (patient reported and medical records) were used to classify patients. Moderate ‘alcohol excess’ was defined as a binary variable indicating a history of consumption greater than 21 units/week for men and 14 units/week for women (1 unit of alcohol=10 mL or 8 g of pure alcohol, an amount the average adult metabolises in 1 hour) but less than 80 g/day for 5 years (the criteria for alcoholic cardiomyopathy). All patients had consumption well below the threshold for alcoholic cardiomyopathy (online supplemental figure 1). The 14-unit and 21-unit thresholds reflect ‘sensible limits’ for alcohol consumption based on UK consensus medical advice^7^ from 1987 to 2016.

10.1136/heartjnl-2021-319418.supp1 Supplementary data

**Table 1:** Definitions of alcohol consumption and moderate alcohol excess in the UK, Europe and North America

Diagnosis	Alcohol consumption	Used in this study	Notes
Alcoholic cardiomyopathy	80 g/day for at least 5 years.	No patients with alcoholic cardiomyopathy were recruited to this study.	–
Moderate alcohol excess according to UK government guidelines 1987–2016	Men: >21 units of alcohol/week. Women: >14 units of alcohol/week.	Yes – the basis of the primary analysis.	UK 1 unit of alcohol=10 mL or 8 g of pure alcohol, an amount the average adult metabolises in 1 hour.
Moderate alcohol excess according to the European Society of Cardiology (ESC) 2016	Men: up to 20 g/day (2 units). Women: up to 10 g/day (1 unit).	Considered in secondary sensitivity analysis; not the basis of the primary analysis.	ESC 1 unit of alcohol=10 g alcohol.
Moderate alcohol excess according to the US Centre for Disease Control/US Dietary Guidelines	Men: >2 standard drinks/day. Women: >1 standard drink/day.	No	US ‘standard drink’=∼12 g alcohol.
Moderate alcohol excess according to the WHO	>2 standard drinks/day.	No	WHO ‘standard drink’=10 g alcohol.

Self-reported weekly alcohol consumption at the time of study enrolment was also documented and evaluated separately, although this did not form the basis of the primary analysis as patients with a history of moderate alcohol excess may have reduced consumption shortly prior to enrolment in this study; therefore, current consumption may not accurately reflect prior consumption. As outlined, individuals meeting criteria for alcoholic cardiomyopathy were not included in the cohort.

### Clinical outcomes

The primary end-point was a composite of cardiovascular mortality, major arrhythmic events and major heart failure events. Cardiovascular mortality and each of the arrhythmic and heart failure composites were predefined secondary end-points. Major arrhythmic events comprised haemodynamically stable and unstable sustained ventricular tachycardia, ventricular fibrillation, appropriate implantable cardiac defibrillator shock and aborted sudden cardiac death. Major heart failure events comprised heart transplantation, left ventricular assist device implantation and unplanned heart failure hospitalisation.

End-points were defined according to the 2014 American College of Cardiology (ACC)/American Heart Association (AHA) definitions for cardiovascular end-points in clinical trials^8^ and the 2006 ACC/AHA/European Society of Cardiology (ESC) guidelines for management of patients with ventricular arrhythmias.^9^ Follow-up data were collected from primary care and hospital medical records and patient questionnaires. Survival status was also identified using the UK Health and Social Care Information Service to ensure no deaths were missed. Death certificates and postmortem reports were obtained where applicable. All primary end-point events were adjudicated by an independent committee of three senior cardiologists with expertise in electrophysiology, heart failure management and clinical trial adjudication. They were blinded to alcohol status, imaging and clinical data. Follow-up time was truncated at 10 years given the reduced number of individuals with follow-up beyond 10 years. Event-free survival was calculated from the date of study entry to the date of the first event in the composite end-point. Data for all patients who were last known to be alive, or who had died after 31 December 2015, were censored on 31 December 2015.

### Statistical methods

Cardiac structure and function was compared between patients with and without a history of moderate alcohol consumption. Continuous data are expressed as median (±IQR) and compared using the Mann-Whitney test. Categorical data are expressed as number and percentages, and compared using Fisher’s exact test. We calculated unadjusted differences in cardiac phenotypes between patients with and without a moderate history of alcohol excess. We then calculated differences after using propensity scores with inverse probability weighting to adjust for differences in age, sex, titin truncating variants and medication use (baseline use of beta blockers, ACE inhibitors, aldosterone antagonists and diuretics). We adjusted for covariates that we judged were likely to be associated with baseline phenotypes but unlikely to have been influenced by prior moderate alcohol consumption.

The Kaplan-Meier method was used to estimate cumulative freedom from the end-point, and the log-rank statistic was used to test the null hypothesis that there was no difference between groups in the probability of an event at any time point.

Cox proportional hazard modelling was used to evaluate the effect of alcohol on the primary endpoint. An optimised baseline model predicting the primary end-point excluding alcohol consumption was built from all clinical, imaging and demographic variables using purposeful variable selection (p value threshold for inclusion was <0.10 for selection of variables from univariable analysis and for exclusion >0.05 for exclusion of variables from multivariable analysis) and then stepwise selection (online supplemental methods). This consisted of left ventricular ejection fraction, left atrial volume, midwall myocardial fibrosis and a prior history of ventricular tachycardia. As a secondary analysis, to explore a potential J-shaped relationship between alcohol intake and cardiovascular outcomes, we calculated HRs for the primary outcome for categories of alcohol consumption at enrolment (0–10 units/week, 10–20 units/week, >20 units/week) relative to no alcohol consumption. All statistical analyses were conducted in the R environment (V.3.3.1).

### Patient and public involvement

No participants were involved in setting the research question or the outcome measures. Patients were involved in the design and implementation of the overall Biobank study through participation in our patient advisory groups. Results from our Cardiovascular Biobank cohort studies are routinely disseminated to study participants through our research group websites and social media outlets. We plan to disseminate these findings to participants and the general public in a press release.

## Results

### Summary of alcohol history in cohort

Among 604 DCM patients, 98 (16%) patients had a history of moderate alcohol excess. Their characteristics are outlined in table 2. Patients with a history of moderate alcohol excess were more likely to be male, but there was no significant difference in age between groups. There was no difference in baseline medication use, history of atrial fibrillation, blood pressure or symptom status at baseline (table 2). Alcohol consumption did not vary by socioeconomic status (p=0.05) (online supplemental table 1).

**Table 2:** Summary of demographics in cohort stratified by alcohol status

	No alcohol excess (n=506)	Moderate alcohol excess (n=98)	P value
**Demographics and comorbidities**			
Age	53.9 (44.0 to 64.9)	54.9 (46.7 to 60.6)	0.99
Sex=male (%)	306 (60.5)	92 (93.9)	**<0.001**
Caucasian=yes (%)	431 (85.2)	93 (94.9)	0.015
LBBB=yes (%)	142 (28.1)	22 (22.4)	0.31
Hypertension=yes (%)	152 (30.0)	28 (28.6)	0.87
Systolic blood pressure (mm Hg)	120 (108 to 134)	122 (111 to 135)	0.57
Diastolic blood pressure (mm Hg)	71 (62 to 81)	75 (66 to 83)	0.05
Atrial fibrillation=yes (%)	123 (24.3)	27 (27.6)	0.52
Diabetes mellitus=yes (%)	66 (13.0)	5 (5.1)	**0.039**
NYHA class (%)			0.49
I	205 (42.8)	47 (49.5)	
II	196 (40.9)	34 (35.8)	
III/IV	78 (16.3)	14 (14.7)	
**Medications**			
Diuretics=yes (%)	224 (44.3)	46 (46.9)	0.71
Beta blocker=yes (%)	353 (69.8)	72 (73.5)	0.54
ACE inhibitor=yes (%)	399 (78.9)	79 (80.6)	0.80
Aldosterone antagonist=yes (%)	181 (35.8)	37 (37.8)	0.80

Data are shown as median (IQR) or numbers (percentages) and compared using the Mann-Whitney test or Fisher’s exact test as appropriate. Moderate alcohol excess is defined as per table 1. No alcohol excess is defined as consumption below these limits including no consumption at all. Bold values indicate p<0.05.

LBBB, left bundle branch block; NYHA class, New York Heart Association functional class.

### Relationship between moderate excess alcohol and cardiac structure

**Figure 1:**
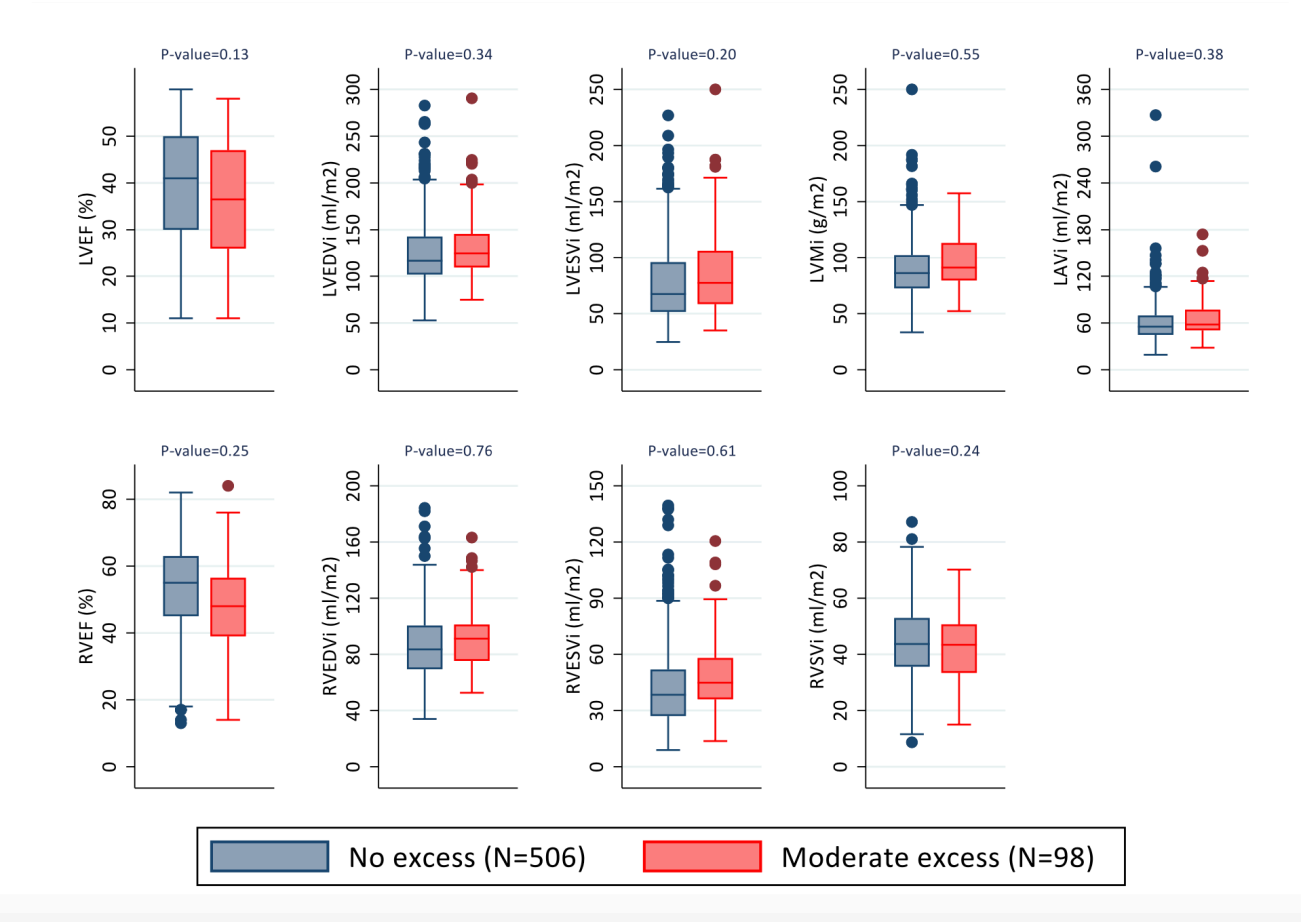
Box plots demonstrating variations in cardiac structure and function in patients with dilated cardiomyopathy stratified by previous alcohol intake. Patients with a history of moderate alcohol excess have lower biventricular function (left and right ventricular ejection fraction (LVEF/RVEF)) and more dilated ventricles (left and right ventricular end diastolic volume, (LVEDVi/RVEDVi); left and right ventricular end systolic volume (LVESVi/RVESVi)), as well as increased left ventricular mass (LVMi) and dilated left atria (left atrial volume (LAVi)). These differences were not robust to adjustment for age, sex and clinical covariates (titin truncating variant status, medication use including beta blockers, ACE inhibitors, aldosterone antagonists and diuretics). Adjusted p values shown.

DCM patients with a history of moderate excess alcohol consumption had a globally more impaired cardiac phenotype compared with patients without moderate alcohol consumption. This was characterised by lower biventricular function and increased chamber dilatation of the left ventricle, right ventricle and left atrium, as well as increased left ventricular hypertrophy (figure 1, unadjusted analysis; table 3). There was no difference in midwall myocardial fibrosis between groups (LGE n=43, 44% in the group with a history of moderate alcohol excess compared with n=170, 34% in no history of moderate excess alcohol group, p=0.07). These findings were not robust to adjustment for age, sex and clinical covariates (table 3). Of note, restricting the analysis to males only did not change the results. Therefore, while patients with moderate alcohol excess are likely to have a worse cardiac structure at presentation, this is largely due to other characteristics, most notably biological sex. There are two clear indicators that this was mostly driven by biological sex. First, sex is the only covariate that is highly imbalanced between those with and without moderate alcohol excess. Second, we formally checked this by adjusting for all other factors, except for sex, which made minimal difference to our results. This suggests that sex specific differences in cardiac structure and function may be mediated by lifestyle factors such as alcohol consumption as 94% of the moderate alcohol excess group were male, compared with 61% of the no alcohol excess group.

**Table 3:** Unadjusted and adjusted analyses of the relationship between moderate alcohol excess and cardiac structure and function

Phenotype	Unadjusted analysis		Adjusted analysis	
	Estimate and 95% CIs	P value	Estimate and 95% CIs	P value
Indexed left atrial volume (mL/m^2^)	5.9 (0.4 to 11.5)	0.035	3.0 (−3.7 to 9.7)	0.38
Indexed left ventricular end diastolic volume (mL/m^2^)	8.3 (0.1 to 16.4)	0.048	4.3 (−4.6 to 13.2)	0.34
Left ventricular ejection fraction (%)	−3.3 (−6.0 to −0.6)	0.017	−3.2 (−7.3 to 1.0)	0.13
Indexed left ventricular end systolic volume (mL/m^2^)	9.4 (1.0 to 17.9)	0.028	6.7 (−3.6 to 17.0)	0.20
Indexed left ventricular mass (g/m^2^)	6.6 (1.5 to 11.7)	0.012	2.4 (−5.5 to 10.2)	0.55
Indexed right ventricular end diastolic volume (mL/m^2^)	5.4 (0.4 to 10.4)	0.034	−1.0 (−7.5 to 5.5)	0.76
Right ventricular ejection fraction (%)	−4.9 (−7.8 to −2.0)	0.0010	−2.7 (−7.3 to 1.9)	0.25
Indexed right ventricular end systolic volume (mL/m^2^)	6.8 (2.3 to 11.3)	0.0030	1.4 (−4.1 to 7.0)	0.61

Adjusted analyses covariates: age, sex, titin truncating variant status, medication use including beta blockers, ACE inhibitors, aldosterone antagonists, and diuretics.

### The effect of moderate excess alcohol consumption on cardiovascular outcomes

Over a median follow-up time of 3.9 years, 78 patients (13%) met the primary composite endpoint, of whom 15 patients had a history of moderate alcohol excess (19%) and 63 patients (81%) did not (p=0.54). The breakdown of the primary endpoint by event and alcohol history is listed in online supplemental tables 2 and 3.

**Figure 2:**
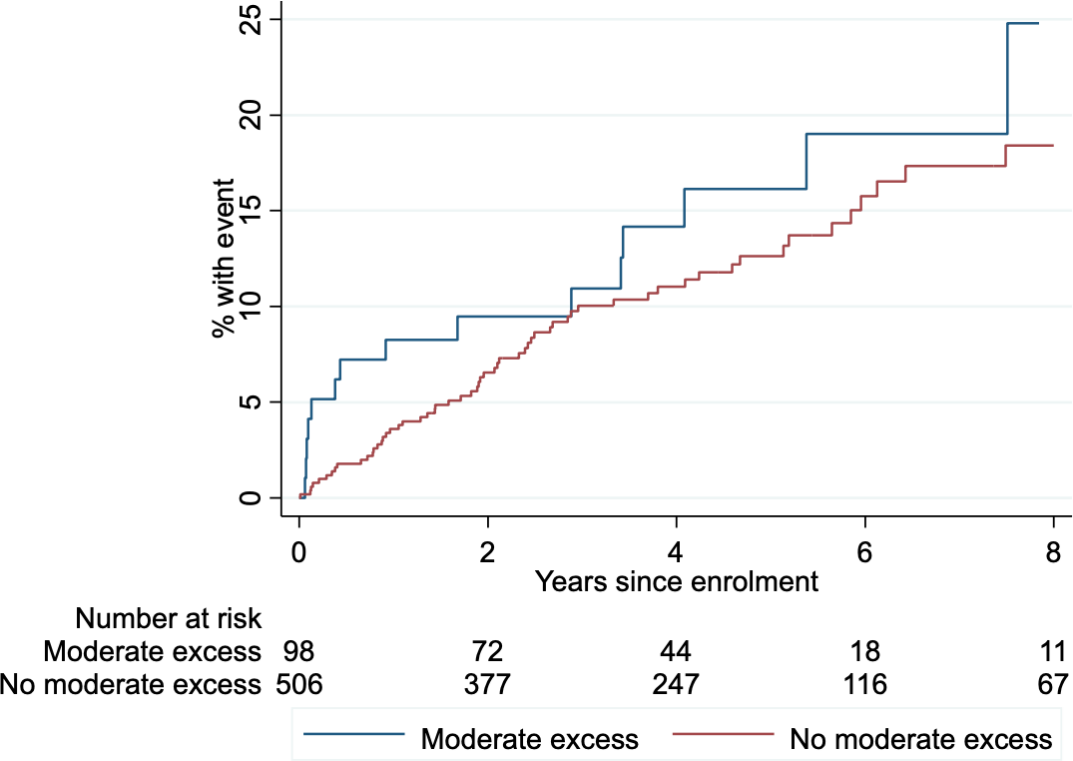
Kaplan-Meier survival curve showing freedom from primary endpoint (composite of cardiovascular death, heart failure events and arrhythmic events) in DCM patients stratified by presence or absence of history of moderate excess alcohol consumption prior to study recruitment. DCM, dilated cardiomyopathy.

There was no difference in freedom from the primary endpoint in DCM patients stratified by alcohol history(figure 2). There was no evidence that a history of moderate alcohol excess had an effect on the composite endpoint in this cohort of patients with DCM (HR 1.29, 95% CI 0.73 to 2.26, p=0.38). Moderate alcohol excess did not have a detectable effect on the secondary endpoints of cardiovascular mortality: HR 0.78 (95% CI 0.23 to 2.60), p=0.68; major heart failure events: HR 0.86 (95% CI 0.39 to 1.91), p=0.71; or major arrhythmic events: HR 2.36 (95% CI 0.97 to 5.71), p=0.057. There was no evidence of a J-shaped relationship between alcohol intake and the primary cardiovascular outcome (online supplemental figure 3).

When adjusted for predefined factors known to predict outcome in this cohort (left ventricular ejection fraction, left atrial volume, prior history of ventricular tachycardia, and midwall fibrosis),^5^ there was no evidence that a history of moderate alcohol excess was associated with the primary endpoint (table 4). On sensitivity analyses, adjustment for age, sex and smoking history did not alter the study findings (online supplemental table 4).

**Table 4:** Adjusted HR for primary composite endpoint evaluating the association between prior alcohol consumption and major cardiovascular outcomes in the study cohort (n=604)

	HR (95% CI)	P value
Indexed left atrial volume (per 10 mL/m^2^)	1.12 (1.07 to 1.17)	0.000004
Presence of midwall fibrosis	2.33 (1.44 to 3.76)	0.0006
Left ventricular ejection fraction (per 10%)	0.7 (0.58 to 0.84)	0.0002
Prior history of sustained VT	3.21 (1.26 to 8.18)	0.01
Moderate alcohol excess prior to recruitment compared with no alcohol excess*	1.02 (0.57 to 1.84)	0.94

*No alcohol excess includes patients with no alcohol consumption and consumption within guideline limits.

VT, ventricular tachycardia.

At the time of study recruitment, 67 of the 98 individuals (68%) with a prior history of moderate alcohol excess self-reported alcohol consumption above the government recommended weekly limits. Among these individuals with *on-going* moderate alcohol excess consumption, there was no evidence for an adverse or beneficial effect of *ongoing* moderate excess alcohol consumption on cardiovascular outcomes (HR for primary endpoint 1.45, 95% CI 0.76 to 2.74, p=0.26).

### Definition of alcohol excess: sensitivity analysis ESC guidelines 2016

In 2016, the ESC updated their dyslipidaemia guidelines outlining that moderate excess alcohol consumption (up to 20 g/day (2 units) for men and 10 g/day (1 unit) for women) was acceptable for those who drink alcoholic beverages.^10^ When DCM patients were recoded according to these limits based on their alcohol consumption at enrolment (moderate excess n=142, no excess n=462), we did not observe that moderate excess alcohol consumption was associated with cardiovascular outcomes (HR for moderate excess compared with no consumption 0.94, 95% CI 0.56 to 1.60, p=0.83; Kaplan-Meier plot in online supplemental figure 2).

## Discussion

These findings demonstrate that moderate excess alcohol consumption is associated with adverse cardiac structure and function in patients with DCM but that these differences are largely due to biological sex. Excessive alcohol consumption is known to be deleterious to cardiovascular health, but the risks and benefits of low to moderate excess alcohol consumption remain an active area of debate. The alcohol consumption by patients in this study was well below the threshold for alcoholic cardiomyopathy. These findings help to inform lifestyle discussions for patients with DCM.

While excessive alcohol consumption is well documented to portend adverse outcomes, there are much less data to guide and inform discussions with patients on moderate levels of consumption. This is a frequently asked question and in need of a greater evidence base to help advise patients. This is the first study to specifically evaluate the effects of a history of moderate excess alcohol consumption in DCM patients and the findings will be informative for both clinicians and patients. In our study, when adjusting for covariates including biological sex, we found that moderate alcohol excess was not associated with adverse cardiac structure. This highlights the sex-specific differences in cardiac structure and function in DCM as well as sex-specific differences in alcohol consumption, as 94% of the cohort with a history of moderate alcohol excess were male compared with 61% of the cohort without such a history. It is plausible therefore that some of the differences in cardiac structure and function seen between men and women with DCM are driven by lifestyle factors such as moderate excess alcohol use.

It is notable that despite adverse cardiac remodelling, moderate excess alcohol consumption was not associated with adverse outcomes. There are a number of possible reasons. One is that moderate excess alcohol consumption may exert a protective effect in terms of cardiovascular risk.^11 12^ It is also possible that the effect of alcohol consumption on outcomes is mediated by an unknown or unmeasured confounder. Insufficient statistical power may also account for these findings; however, we would expect to see evidence of a large effect on outcome (either beneficial or adverse) in this cohort size; therefore, this is unlikely to be the entire explanation. This study prompts further research to explore the reasons behind this novel finding.

A J-shaped or U-shaped relationship between alcohol consumption and total mortality has been well documented. There has been conflicting evidence for the role of low to moderate excess alcohol consumption in cardiovascular disease more generally^13 14^ and specifically with respect to heart failure phenotypes. A large study of ~6000 individuals followed up for ~10 years in the Australian National Blood pressure study found no association between alcohol consumption and risk of incident heart failure in either men or women.^15^ In contrast, in an Italian study of ~22 000 individuals followed for ~8 years, light to moderate alcohol consumption (1–4 drinks/day) was associated with a lower risk for heart failure, with a maximal 22% risk reduction for individuals consuming 20 g alcohol per day.^12^

A lack of international standardisation of alcohol intake and consensus definitions of low, moderate and high consumption makes between study comparisons more challenging. The WHO guidelines on alcohol define one standard drink to be 10 g of pure ethanol and recommend no more than two standard drinks per day,^16^ but these definitions are not universally adopted among all countries.^17^ In addition, there is compelling evidence that there may not be a ‘safe’ threshold for alcohol consumption in terms of cardiovascular risk, and cardiovascular risks may increase with alcohol consumption at levels lower than considered in this study.^18^ In a pooled analysis of almost 600 000 individuals without cardiovascular disease, alcohol consumption <12.5 units/week was associated with the lowest risk of cardiovascular death. However, for other cardiovascular outcomes including heart failure (as well as stroke, coronary artery disease excluding myocardial infarction and fatal hypertensive disease), there was no clear risk thresholds below which lower alcohol consumption stopped being associated with lower disease risk.^18^

Midwall fibrosis remained a strong predictor of outcome in DCM in our study, irrespective of history of alcohol excess. Interestingly, although there was no statistically significant difference in the presence of midwall fibrosis between both groups, more individuals with a history of higher alcohol consumption had midwall fibrosis compared with individuals without a history of alcohol excess. In contrast, a previous CMR study of 165 light to moderate drinkers without pre-existing cardiac disease suggested that these individuals had less diffuse myocardial fibrosis (lower T1 times) compared with non drinking controls.^19^ The differences may be due to the presence of overt cardiomyopathy in this cohort.

There are some potential limitations to this analysis. With regards to current alcohol consumption, we only have the data recorded at one time point at study recruitment and do not know how patients’ consumption may have changed over the time course of the study follow-up. This may have affected the outcome in a time-varying manner, which is not accounted for in the current study design. In real-world settings, however, guidance is similarly based on reported consumption, so this study takes a pragmatic approach. The effect of ongoing consumption should be addressed in future prospective studies. In addition, although all the analyses suggest that it is unlikely that alcohol use is associated with a much lower risk of adverse cardiovascular outcomes, we cannot definitively exclude that consumption within guideline limits is not protective or that that the risk of moderate excess alcohol consumption is not increased in the current sample size and follow-up period. Finally, the majority of studies evaluating alcohol and cardiovascular health are observational, including this one, and despite optimal study design and statistical analysis they are subject to the effect of confounders. Due to ethical considerations, randomised controlled trials in this area are limited, although one is currently in progress (Moderate Alcohol and Cardiovascular Health Trial; NCT03169530). Globally, alcohol consumption is an established risk factor for many chronic diseases and contributes to an increase in disease burden.

## Conclusion

Excessive alcohol consumption is known to be deleterious to cardiovascular health, but the risks and benefits of low to moderate alcohol consumption remains an active area of debate. We demonstrate that moderate excess alcohol consumption is associated with adverse cardiac structure and function in patients with DCM, though this was driven by the preponderance of men in the alcohol excess group suggesting that sex-specific differences in cardiac structure in DCM may be driven by lifestyle factors such as alcohol use. This study adds to the growing body of evidence refuting a substantial cardioprotective effect of moderate alcohol consumption. We were unable to detect a large beneficial or large adverse effect on medium term outcomes. Larger studies are needed to evaluate for potentially safe or harmful thresholds of alcohol consumption among patients with DCM.

**Key messages**

**What is already known on this subject?**

- Marked excess alcohol consumption (>80 g/day for 5 years) is associated with alcoholic cardiomyopathy.
- The effects of moderate excess alcohol consumption on cardiac structure and function is not known.
- There is no clear evidence base to guide lifestyle advice on alcohol consumption for patients with dilated cardiomyopathy.

**Whatdoes this study add?**

- Patients with dilated cardiomyopathy who have a history of moderate excess alcohol consumption (>14 units/week for women, >21 units/week for men) have increased biventricular dilatation and biventricular impairment as well as left atrial dilation and more hypertrophy compared with patients without moderate excess alcohol consumption.
- After adjusting for biological sex, moderate alcohol excess was not associated with adverse cardiac structure suggesting that lifestyle factors such as moderate excess alcohol use may contribute to the sex-specific differences in cardiac structure in dilated cardiomyopathy.
- This study did not find either a beneficial or large adverse effect on cardiovascular outcomes.

**How might this impact on clinical practice?**

- The alcohol consumption by patients in this study was well below the threshold for alcoholic cardiomyopathy. This study suggests that particularly for men with dilated cardiomyopathy, even moderate alcohol excess is associated with adverse cardiac structure and function.
- These findings help to inform lifestyle discussions for patients with dilated cardiomyopathy.

## Data Availability

Data are available on reasonable request. The data and analysis methods that support the findings of this study are available from the corresponding author, (UT) on reasonable request. Data will be shared after review and approval by our Biobank scientific board, and terms of collaboration will be reached together with a signed data access agreement.
