# Supplementary material for: Moderate excess alcohol consumption and adverse cardiac remodelling in dilated cardiomyopathy
Source: Heart. 2021 Aug 11;108(8):619–25. doi: 10.1136/heartjnl-2021-319418 (PMC8961767; doi:10.1136/heartjnl-2021-319418)
Supplement: Supplementary data [file heartjnl-2021-319418supp001.pdf]

Moderate alcohol consumption is associated with adverse cardiac remodelling in dilated cardiomyopathy

Tayal et al, 2021

Supplementary files

Supplementary Methods

Baseline Cox model to predict primary outcome

An optimized baseline model predicting the primary end-point was built using Cox proportional hazard modeling evaluating clinical, imaging and demographic variables. As the primary analysis was to evaluate alcohol consumption and outcomes, the baseline model was built without inclusion of alcohol data, using purposeful variable selection (Hosmer and Lemeshow). The p value threshold for inclusion was <0.10 (for selection of variables from univariable analysis) and for exclusion >0.05 (for exclusion of variables from multivariable analysis).

The variables that were significant at a threshold of p<0.10 were considered for inclusion in full model building. At this stage, any variables with potential for inclusion in the next stage of model building were reviewed for co-linearity. The final variables included in the full model, prior to reverse stepwise selection, were left ventricular ejection fraction (LVEF), right ventricular ejection fraction, mid-wall fibrosis late gadolinium enhancement, indexed left ventricular end systolic volume, indexed left atrial volume, a family history of dilated cardiomyopathy, a history of sustained ventricular tachycardia, NYHA class, a history of hypertension, diuretic use, and ACE inhibitor use. From this, stepwise selection was performed, removing the least significant variable in turn until only significant variables were remaining (p<0.05).

This left the baseline model of left ventricular ejection fraction, indexed left atrial volume, mid-wall fibrosis late gadolinium enhancement and a history of sustained ventricular tachycardia. Each previously discarded variable was added to the model in turn. None of these variables became significant on addition to the baseline model, nor did their inclusion affect the hazard ratios or p value for the remaining variables. As left and right ventricular ejection fraction are correlated, the forced inclusion of both variables was tested. The model with just left ventricular ejection fraction had a marginally lower Akaike information criterion (AIC) compared to the model with just right ventricular ejection fraction. In the model with both right and left ventricular ejection fraction, neither variable remained significant. Therefore left ventricular ejection fraction only was retained. Left ventricular end systolic volume (LVESVi), left ventricular end diastolic volume (LVEDVi) and left ventricular mass (LVMi) were not included in the multivariate model to avoid colinearity, but separate models substituting these variables individually for LVEF showed that they too were independently associated with the primary outcome, thought with smaller effect sizes and higher p values (HR and 95% CI for primary end-point: LVESVi per 10mL/m^2= 1.09, 1.03 to 1.16, p=0.001; LVEDVi per 10mL/m^2= 1.07, 1.01 to 1.13, p=0.02). LVMi was not predictive of the primary outcome (HR and 95% CI; LVMi per 10g/m^2 = 1.04, 0.94-1.15, p=0.42). The final baseline model consisted of LVEF, LAVi, LGE and ventricular tachycardia. The model was tested and there was no violation of the proportionality assumption, either for individual variables or the global model.

Supplementary Results

Alcohol consumption at enrollment

Data on self-reported weekly alcohol consumption at the time of enrollment was available in 591 participants. The majority of the cohort reported no weekly alcohol consumption (n=301, 51%, Supplementary Figure 1). Six individuals reported weekly consumption over 70 units but no patients met criteria for alcoholic cardiomyopathy.

At the time of study recruitment, 67 of the 98 individuals (68%) with a prior history of moderate alcohol excess self-reported alcohol consumption above the government recommended weekly limits. Amongst these individuals with on-going moderate alcohol excess consumption, there was no evidence for an adverse or beneficial effect of ongoing moderate alcohol consumption on cardiovascular outcomes (HR for primary endpoint 1.45, 95% CI 0.76 to 2.74, p= 0.26).

Overall, 86 individuals (15%) in the total cohort (irrespective of history of moderate alcohol excess prior to recruitment) self reported weekly alcohol consumption in excess of UK government guidelines at the time of study recruitment. Compared to individuals who did not consume alcohol and those individuals who drank within government limits, there was no difference in outcome (Supplementary Figure 3, Supplementary Table 4). In addition, there was no relationship between alcohol consumption categorised as 0-10 units/week, 10-20 units/week , or >20 units/week compared to no alcohol consumption at enrollment and cardiovascular outcomes (Supplementary Figure 4).

Tayal et al, 2021

Supplementary Tables

Supplementary Table 1: Alcohol consumption by socioeconomic status. Socio-economic status is estimated using the Index of Multiple Deprivation quintile (IMD). Groups are compared using the Fisher's exact test. Whilst IMD quintile 4 had a higher proportion of patients with a history of moderate alcohol excess, overall there was no significant difference between groups (p=0.05) suggesting that in this cohort alcohol consumption was not affected by socio-economic status.

IMD quintile	**1**	**2**	**3**	**4**	**5**
No alcohol excess	39	96	91	106	132
Alcohol excess	7 (15%)	12 (11%)	16 (15%)	35 (25%)	22 (14%)

Supplementary Table 2: Table showing the number of patients meeting each end-point. *Cardiovascular death included 18 patients with heart failure death, 3 patients with sudden cardiac death, and 1 patient each with acute myocardial infarction, cerebrovascular accident and cardiovascular –other.

	**Primary composite**	**Cardiovascular death***	**Arrhythmic secondary**	**Heart Failure secondary**
Yes	78	24	24	50
No	526	580	580	554

Note: The number of primary composite events does not equal the sum of the components (cardiovascular death, arrhythmic outcome, heart failure outcome) because some patients may experience more than one type of outcome during follow up.

Tayal U, *et al. Heart* 2022; 108:619–625. doi: 10.1136/heartjnl-2021-319418

Tayal et al, 2021

Supplementary Table 3: Composition of events leading to primary composite end-point. Patients were censored at first event.

CV death	Heart Failure hospitalization	Cardiac transplant	LVAD	Stable sustained VT	Unstable sustained VT	Aborted sudden cardiac death	Appropriate ICD activation	Ventricular fibrillation	Moderate alcohol excess	Number of patients with this combination of events (n=78)
No	No	No	No	No	No	Yes	Yes	Yes	TRUE	1
No	No	No	No	Yes	No	No	No	No	TRUE	3
No	No	No	No	Yes	No	Yes	Yes	No	TRUE	1
No	No	No	No	Yes	Yes	Yes	Yes	No	TRUE	1
No	Yes	No	No	No	No	No	No	No	TRUE	5
No	Yes	Yes	No	No	No	No	No	No	TRUE	1
Yes	No	No	No	No	No	No	No	No	TRUE	2
Yes	Yes	No	No	Yes	No	No	No	Yes	TRUE	1
No	No	No	No	Yes	No	No	No	No	FALSE	4
No	No	No	No	Yes	No	Yes	Yes	No	FALSE	2
No	No	No	No	Yes	Yes	No	No	No	FALSE	1
No	No	No	No	Yes	Yes	Yes	Yes	No	FALSE	2
No	No	No	No	Yes	Yes	Yes	Yes	Yes	FALSE	1
No	No	No	Yes	No	No	No	No	No	FALSE	1
No	No	Yes	No	No	No	No	No	No	FALSE	2
No	No	Yes	Yes	No	No	No	No	No	FALSE	1
No	Yes	No	No	No	No	No	No	No	FALSE	19
No	Yes	No	No	No	Yes	Yes	Yes	Yes	FALSE	1
No	Yes	No	No	Yes	No	No	No	No	FALSE	1
No	Yes	No	Yes	No	No	No	No	No	FALSE	2
No	Yes	No	Yes	No	Yes	Yes	Yes	No	FALSE	1
No	Yes	Yes	No	No	No	No	No	No	FALSE	2
No	Yes	Yes	No	Yes	Yes	Yes	Yes	No	FALSE	1
No	Yes	Yes	Yes	No	No	No	No	No	FALSE	1
Yes	No	No	No	No	No	No	No	No	FALSE	10
Yes	No	No	Yes	Yes	Yes	Yes	Yes	No	FALSE	1
Yes	Yes	No	No	No	No	No	No	No	FALSE	8
Yes	Yes	No	No	Yes	No	Yes	Yes	No	FALSE	1
Yes	Yes	No	Yes	Yes	No	Yes	No	Yes	FALSE	1

Tayal U, *et al. Heart* 2022; 108:619–625. doi: 10.1136/heartjnl-2021-319418

Supplementary Table 4: Sensitivity analyses adjusting for additional covariates.

Covariates included in model	Hazard ratio for moderate alcohol consumption compared to no alcohol excess**	P-value
Adjusted for LAVi, LVEF, VT and LGE only	1.02 (0.57 to 1.84)	0.94
Adjusted for LAVi, LVEF, VT and LGE and*		
Smoking history	1.04 (0.57 to 1.94)	0.88
Age	1.04 (0.58 to 1.86)	0.91
Sex	1.14 (0.62 to 2.10)	0.68
Smoking, age, sex	1.10 (0.61 to 2.0)	0.73

*Each statistical model contains 5 covariates, LAVi, LVEF, VT and LGE, plus the covariate listed in the table
**No alcohol excess includes patients with no alcohol consumption and consumption within guideline limits.

Smoking history: Of the cohort of 604 patients, 59 were smokers at the time of enrollment, 152 were ex smokers, 333 were non smokers and there was no recorded data for the remaining 60 patients.

Tayal et al, 2021

Supplementary Figures

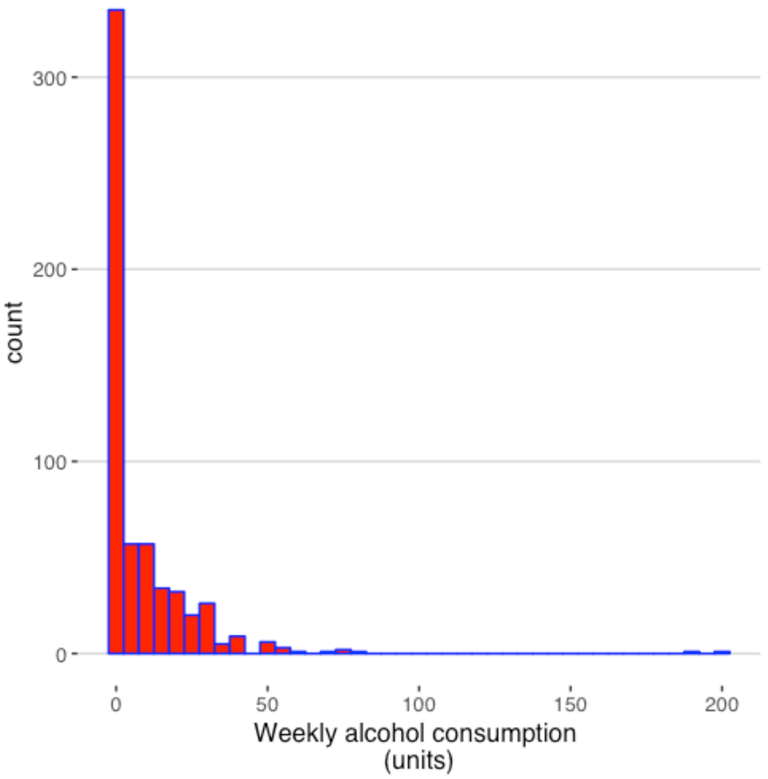

Supplementary Figure 1: Histogram of distribution of weekly self reported alcohol consumption in cohort at time of study recruitment. No patient met criteria for alcoholic cardiomyopathy (sustained alcohol consumption of >80g/day for 5 years).

Tayal U, *et al. Heart* 2022; 108:619–625. doi: 10.1136/heartjnl-2021-319418

Tayal et al, 2021

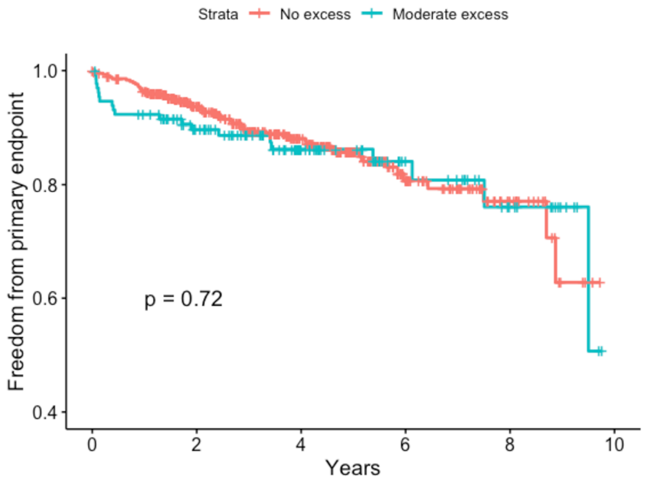

Supplementary Figure 2: Sensitivity analysis – ESC guidelines. Kaplan Meier survival curve showing freedom from primary endpoint (composite of cardiovascular death, heart failure events, and arrhythmic events) in DCM patients stratified by current self reported weekly alcohol consumption at the time of study recruitment in line with 2016 ESC guidelines on recommended alcohol intake.

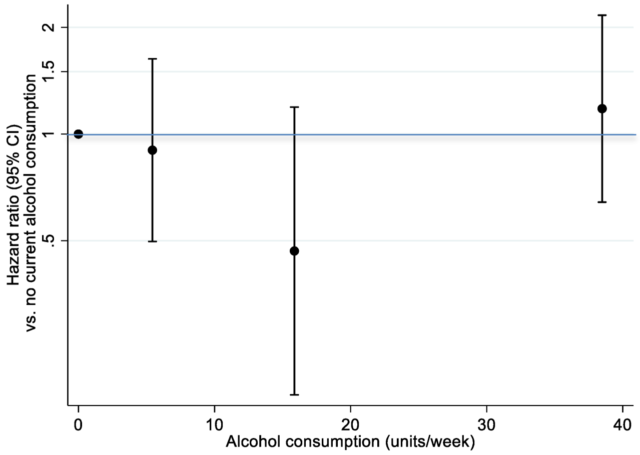

Supplementary Figure 3: Relationship between alcohol consumption at enrollment and the primary endpoint. There is no significant relationship between alcohol consumption and outcomes in this cohort. Blue line indicates hazard ratio of 1.
